# Trends of Healthy Lifestyles Among Adolescents: An Analysis of More Than Half a Million Participants From 32 Countries Between 2006 and 2014

**DOI:** 10.3389/fped.2021.645074

**Published:** 2021-05-25

**Authors:** Priscila Marconcin, Margarida G. Matos, Andreas Ihle, Gerson Ferrari, Élvio R. Gouveia, Marcos López-Flores, Miguel Peralta, Adilson Marques

**Affiliations:** ^1^Faculty of Human Kinetics, University of Lisbon, Lisbon, Portugal; ^2^Instituto de Saúde Ambiental, Universidade de Lisboa, Lisboa, Portugal; ^3^Center for the Interdisciplinary Study of Gerontology and Vulnerability, University of Geneva, Geneva, Switzerland; ^4^Swiss National Centre of Competence in Research LIVES–Overcoming Vulnerability: Life Course Perspectives, Geneva, Switzerland; ^5^Department of Psychology, University of Geneva, Geneva, Switzerland; ^6^Escuela de Ciencias de la Actividad Física, el Deporte y la Salud, Universidad de Santiago de Chile (USACH), Santiago, Chile; ^7^Departamento de Educaçao Física e Desporto, Universidade da Madeira, Funchal, Portugal; ^8^Interactive Technologies Institute, Laboratory of Robotics and Engineering Systems, Funchal, Portugal; ^9^Faculty of Health Sciences, Universidad Isabel I, Burgos, Spain; ^10^Centro Interdisciplinar de Estudo da Performance Humana, Faculty of Human Kinetics, University of Lisbon, Lisbon, Portugal

**Keywords:** epidemiologic research design, health lifestyle, adolescents, vulnerability, health policies

## Abstract

The purpose of this study was to provide data regarding the prevalence and trends of adolescents' healthy lifestyles from 32 countries between 2006 and 2014 by sex and age interval. The data used in the present study were derived from the Health Behavior in School-aged Children (HBSC) 2006, 2010, and 2014 international database. Healthy lifestyle was assessed using the combination of daily physical activity, daily fruit and vegetable consumption, <2 h daily on screen-based behaviors, abstinence from alcohol, and abstinence from tobacco products. Healthy lifestyle measures were based on self-report. The final sample comprised 519,371 adolescents (aged between 10 and 16 years old). The prevalence of healthy lifestyle behaviors increased between 2006 and 2014. The healthy lifestyle score worsened with advancing age for boys and girls. Comparing countries, for boys, the highest values were observed in adolescents from Ireland (5.2%, 95% CI: 3.9, 6.4), and for girls, the highest values were observed in adolescents from Iceland (4.2%, 95% CI: 3.6, 4.7). The present study showed a slight trend to an improved healthy lifestyle among adolescents, although much more has to be done. A joint effort from multiple areas of knowledge must be made to improve adolescent health policies, since lifestyles in adolescence play an important role for the development of vulnerability and health in later life.

## Introduction

Adolescence corresponds to the period between the ages of 10 and 19 years (1). This is a critical period for individuals to establish enduring healthy behaviors (2). Important health-related behaviors initiate at this period and track into adult life, thereby playing an important role for vulnerability in later life (3). For example, higher amounts of alcohol consumption in adolescence increased the odds of being a heavy drinker in the early years of adulthood (4). The World Health Organization (5) puts out that the four major risk factors for chronic disease during adulthood are alcohol consumption, poor nutrition and diet, physical inactivity, and tobacco consumption. This could mean that a healthy lifestyle in adolescence can minimize chronic diseases in adulthood.

For children and adolescents, there is strong evidence that the main health behaviors associated with health status and quality of life are doing physical activity daily, spending <2 h of screen-based sedentary behaviors, eating fruit and vegetables daily, and abstinence from alcohol and tobacco (6, 7).

Epidemiological studies have shown a positive association between high levels of physical activity and eating healthy foods with good health and health-related quality of life (8, 9). A healthy lifestyle is also significantly related to not having subjective health complaints (6). On the other hand, alcohol use, drug use, unprotected sex, sleep duration, and smoking are all considered important predictors of non-communicable diseases during adolescence (10).

Previous studies, using data from the Health Behavior in School-aged Children (HBSC), have reported the prevalence of adolescent's healthy lifestyles (6, 7). Those studies evaluated healthy lifestyle as a composite measure of doing physical activity daily, spending <2 h of screen-based sedentary behaviors, eating fruit and vegetables daily, and abstinence from alcohol and tobacco. Those studies found that the prevalence decreases significantly with ages between 11 and 15 years, and on average, it does not exceed 2%. Also, girls present worse values compared to boys. However, only data from one survey (2010 and 2014) were used and thus did not focus on the prevalence and trends of adolescents' healthy lifestyles. Monitoring adolescents' healthy lifestyle prevalence and trends is essential to support interventions aiming to promote a healthy lifestyle, such as increasing physical activity, and diminish sedentary behavior, improve food handling, and prevent alcohol and tobacco consumption. This is particularly important to promote a healthy lifestyle in adulthood and consequently prevent chronic conditions in later life (3, 11). Therefore, targeting this important issue, the present study aimed to investigate the prevalence and trends of adolescents' healthy lifestyles from the HBSC 2006, 2010, and 2014 international database. Here, 32 countries participated in HBSC 2006, 2010, and 2014; most countries were members of the European Union (EU), with the exception of Canada, Israel, Ireland, Macedonia, Russia, Scotland, and Wales. Only Canada is not from Europe. Most are high-income countries with the exception of Macedonia and Russia, which are middle-income countries.

## Methods

### Participants and Procedures

The data used in the present study were derived from the HBSC international database, from 2006, 2010, and 2014. Data are available at http://www.hbsc.org. The HBSC is a cross-national study to gain insights into young peoples' well-being, health behaviors, and social context. HBSC is a school-based survey, with data collected through self-completion questionnaires administered in the classroom (12). More details about the methods and design of the HBSC are described elsewhere (12, 13). Each participant country is responsible for researching under their ethical guidelines; consequently, consent to carry out the research was given by school administrators in each country. Besides, consent to participate was also sought from legal guardians by written informed consent, and adolescents provided assent, since participation was anonymous.

The specific population targeted for sampling was young people attending school aged 11, 13, and 15 years. A minimum of 95% of the eligible target population should be within the sample frame. Countries may choose to stratify their samples to ensure representation by geographic location, ethnic group, or school type (12). The present study includes adolescents (aged 10–16 years) who reported physical activity levels, screen-based sedentary behaviors, eating fruit and vegetables, alcohol consumption, and tobacco use. The final sample comprised 519,371 participants in total, that is, 168,179 participants (80,881 boys and 87,298 girls) from 2006, 176,321 participants (85,195 boys and 91,126 girls) from 2010, and 174,871 participants (84,781 boys and 90,090 girls) from 2014.

Adolescents from 32 countries (Austria, Belgium, Canada, Croatia, Czech Republic, Denmark, England, Estonia, Finland, France, Germany, Greece, Hungary, Iceland, Ireland, Israel, Italy, Latvia, Luxembourg, Macedonia, Netherlands, Norway, Poland, Portugal, Romania, Russia, Scotland, Slovakia, Slovenia, Sweden, Switzerland, and Wales) participated in the present study. Most are middle- and high-income countries.

## Measures

### Sociodemographic Characteristics

Participants reported their sex, age, details about their family, and who they lived with. Socioeconomic status was assessed by the Family Affluence Scale (FAS), a valid measure for adolescents' wealth, and a proxy of socioeconomic status (14). The scale combines responses of four items: family car ownership, bedroom for themselves, holidays, and the number of computers at home.

### Healthy Lifestyle Behaviors and Healthy Lifestyle Composite Score

Physical activity was assessed by the answer about “the number of days over the past week during which they were physically active for a total of at least 60 min per day” into an 8-point scale (0 = none, 7 = daily). Later, the answers were dichotomized, according to the physical activity guidelines (15), into 0 = less than daily and 1 = daily. Screening time was assessed consonant how many hours per day they spend watching television, playing videogames, and using the computer. Answers were dichotomized into ≥2 h/day and <2 h/day (16). Adolescents were asked about how many times they eat fruit and vegetables per week. The options were “never,” “less than once a week,” “once a week,” “2–4 days a week,” “5–6 days a week,” “once every day,” and “several times every day.” Later, answers were dichotomized into daily and less than daily according to international recommendations (17). Alcohol consumption was assessed by the question “How often do you drink alcohol (beer, wine, and liquor/spirits)?” For each alcoholic drink, response options were “never,” “rarely,” “every month,” “every week,” and “every day.” The answers were dichotomized into never drinking and drinking (independently of frequency) because there is no safe amount for alcohol consumption for adolescents (18). A similar question was done about tobacco (“How often do you smoke tobacco at present?”). Response options were “every day,” “at least once a week, but not every day,” “less than once a week,” or “never.” Answers were dichotomized into current smoker (regularly or sometimes) and non-smoker.

Adolescents scored one point for achieving each of the following healthy lifestyle categories: (a) daily physical activity, (b) daily consumption of fruit and vegetables, (c) spending <2 h daily on screen-based sedentary behaviors, (d) never drinking, and (e) never smoking. Thus, the healthy lifestyle score ranged from 0 to 5; only a score of 5 represented a healthy lifestyle. The composite score of a healthy lifestyle was used in previous studies (6, 7).

### Statistical Analyses

Descriptive statistics were performed to characterize the sample. For the prevalence of each healthy lifestyle behavior and the composite score, the percentage and 95% confidence interval (CI) were calculated. Significant differences were analyzed by overlapping 95% CI (19). Data analysis was performed using IBM SPSS Statistics 22 (SPSS Inc., IBM Corp., Armonk, New York, NY, USA).

## Results

Table 1 presents the adolescents' characteristics in 2006, 2010, and 2014. Overall, most adolescents lived with their mother and father. The values of the family affluence scale for all data analyzed stand out, where most adolescents belonged to families with medium and high economic status. Only 9.2% (95% CI: 9.0, 9.3) in 2006, 5.4% (95% CI: 5.3, 5.5) in 2010, and 7.1% (95% CI: 7.0, 7.2) in 2014 belonged to a family with low economic status.

**Table 1:** Participants' characteristics.

	**Year 2006**	**Year 2010**	**Year 2014**
**Characteristics**	**M or %** **(95% CI)**	**M or %** **(95% CI)**	**M or %** **(95% CI)**
	***n* = 168,179**	***n* = 176,321**	***n* = 174,871**
**Sex**			
Boys	48.1 (47.9, 48.3)	48.3 (48.1, 48.6)	48.5 (48.2, 48.7)
Girls	51.9 (51.7, 52.1)	51.7 (51.4, 51.9)	51.5 (51.3, 51.8)
Age (years)	13.63 (13.63, 13.64)	13.61 (13.60, 13.62)	13.15 (13.15, 13.16)
**Age interval**			
10–12 years	32.7 (32.4, 32.9)	31.5 (31.3, 31.8)	32.1 (31.8, 32.3)
13–14 years	34.4 (34.1, 34.6)	34.9 (34.7, 35.1)	35.5 (35.3, 35.7)
15–16 years	33.0 (21.7, 33.2)	33.5 (33.3, 33.8)	32.5 (32.3, 32.7)
**Live with**			
Only mother	19.5 (19.4, 19.8)	20.1 (19.9, 20.3)	19.3 (19.1, 19.5)
Only father	2.6 (2.5, 2.6)	3.0 (2.9, 3.0)	2.8 (2.7, 2.9)
Mother and father	73.7 (73.5, 73.9)	74.1 (73.8, 74.3)	73.4 (73.2, 73.6)
Other people	4.2 (4.1, 4.3)	2.8 (2.8, 3.0)	4.5 (4.4, 4.6)
**Have brothers or sisters**			
No	14.5 (14.3, 14.6)	16.9 (16.6, 17.0)	17.5 (17.3, 17.7)
1–2 brothers/sisters	67.6 (67.3, 67.8)	66.6 (66.6, 67.1)	64.7 (64.4, 64.9)
≥3 brothers/sisters	18.0 (17.8, 18.2)	16.5 (16.2, 16.6)	17.8 (17.6, 18.0)
**FAS**			
Low	9.2 (9.0, 9.3)	5.4 (5.3, 5.5)	7.1 (7.0, 7.2)
Medium	43.4 (43.1, 43.6)	35.1 (34.9, 35.3)	36.0 (35.8, 36.2)
High	47.4 (47.2, 47.7)	59.5 (59.3, 59.7)	56.9 (56.7, 57.2)

*FAS, family affluence scale; M, median; CI, confidence interval*.

The prevalence of healthy lifestyle behaviors by sex and age is shown in Table 2. Overall, between 2006 and 2014, the prevalence of having a healthy lifestyle increased. Between 2006 and 2010, there was no significant difference in the prevalence of healthy lifestyle, but between 2010 and 2014, a significant difference was observed for all age intervals for boys and girls. The trend was to be healthier in 2014. The healthy lifestyle score worsened with advancing age for boys and girls. The highest value for a healthy lifestyle was for girls between 10 and 12 years in 2014 (3.8%, 95% CI: 3.6, 4.0), and the lowest value was for girls between 15 and 16 years in 2006 (0.3%, 95% CI: 0.3, 0.4). Boys presented the same tendency—the highest value was between 10 and 12 years in 2014 (3.2%, 95% CI: 3.0, 3.4), and the lowest value was between 15 and 16 years in 2006 (0.4%, 95% CI: 0.3, 0.4) and in 2010 (0.4%, 95% CI: 0.4, 0.5). Regarding the number of behaviors, 2006 was the year of more adolescents presenting none of the behaviors, and 2014 was the year with fewer adolescents presenting none of the healthy behaviors. A significant difference between 2010 and 2014 was observed for all age intervals for boys and girls. For boys, the most difficult behaviors to reach the recommended value were to eat fruits and vegetables daily and spend <2 h on screen. The lowest value was in 2010 when just 12.6% (95% CI: 12.3, 13.0) of boys spent <2 h on screen. For girls, the most difficult lifestyle behavior to reach was physical activity 60 min daily. The worst values for physical activity every day were between 15 and 16 years old in 2010—only 9.5% (95% CI: 9.2, 9.9) engaged in physical activity every day.

**Table 2:** Healthy behaviors' prevalence in 2006, 2010, and 2014 per sex and age interval.

	**Boys**								
	**10–12 years**			**13–14 years**			**15–16 years**		
	**% (95% CI)**			**% (95% CI)**			**% (95% CI)**		
**Healthy lifestyle behavior**	**2006**	**2010**	**2014**	**2006**	**2010**	**2014**	**2006**	**2010**	**2014**
Physical activity every day	29.1 (28.6, 29.7)	26.9 (26.3, 27.4)	29.2 (28.8, 29.8)	24.7 (24.2, 25.2)	23.7 (23.2, 24.2)	24.3 (23.8, 24.7)	19.3 (18.8, 19.7)	19.0 (18.5, 19.4)	20.4 (20.0, 20.9)
Screen time <2 h/day	25.8 (25.2, 26.3)	24.1 (23.6, 24.6)	33.1 (32.5, 33.7)	17.1 (16.6, 17.5)	15.6 (15.2, 16.0)	20.2 (19.7, 20.6)	15.4 (15.0, 15.9)	12.6 (12.3, 13.0)	16.1 (15.6, 16.5)
Fruit/vegetable every day	19.4 (18.9, 19.9)	22.3 (21.8, 22.8)	23.8 (23.3, 24.3)	17.3 (16.8, 17.7)	18.7 (18.3, 19.1)	19.4 (19.0, 19.9)	14.8 (14.4, 15.2)	16.2 (15.7, 16.6)	18.0 (17.5, 18.4)
Not drink	62.3 (61.7, 62.8)	71.0 (70.5, 71.6)	74.5 (73.9, 75.0)	45.7 (45.1, 46.3)	54.6 (54.1, 55.2)	61.1 (60.6, 61.7)	24.1 (23.6, 24.6)	29.6 (29.1, 30.2)	37.5 (36.9, 38.0)
Not smoke	96.9 (96.7, 97.1)	97.1 (96.9, 97.3)	98.0 (97.8, 98.2)	90.3 (89.9, 90.6)	90.8 (90.5, 91.1)	94.1 (93.9, 94.4)	75.8 (75.3, 76.3)	76.4 (75.9, 76.9)	83.8 (83.4, 84.2)
**Number of behaviors**									
None	3.4 (3.2, 3.6)	1.1 (0.9, 1.2)	0.6 (0.5, 0.7)	7.5 (7.2, 7.8)	4.6 (4.4, 4.8)	2.6 (2.4, 2.8)	14.4 (14.1, 14.9)	14.2 (13.7, 14.6)	9.0 (8.7, 9.4)
1. Behavior	21.0 (20.6, 21.5)	14.3 (13.9, 14.7)	10.8 (10.4, 11.2)	31.3 (30.8, 31.9)	24.6 (24.1, 25.1)	20.3 (19.9, 20.8)	40.9 (40.3, 41.5)	37.2 (36.7, 37.8)	33.0 (32.5, 33.6)
2. Behavior	38.0 (37.4, 38.5)	41.9 (41.4, 42.5)	38.6 (38.0, 39.2)	37.1 (36.5, 37.7)	42.1 (41.6, 42.7)	43.2 (42.6, 43.7)	30.4 (29.8, 30.9)	33.1 (32.5, 33.6)	37.2 (36.6, 37.8)
3. Behavior	26.2 (25.6, 26.7)	29.7 (29.2, 30.3)	32.6 (32.0, 33.1)	18.1 (17.8, 18.7)	21.2 (20.7, 21.6)	24.3 (23.8, 24.8)	11.2 (10.8, 11.5)	12.1 (11.7, 12.4)	15.5 (15.1, 15.9)
4. Behavior	9.4 (9.1, 9.8)	10.8 (10.4, 11.2)	14.2 (13.8, 14.6)	5.1 (4.9, 5.4)	6.6 (6.3, 6.9)	8.3 (8.0, 8.6)	2.7 (2.5, 2.9)	3.0 (2.8, 3.2)	4.6 (4.3, 4.8)
5. Behavior (healthy lifestyle)	2.0 (1.8, 2.2)	2.2 (2.0, 2.3)	3.2 (3.0, 3.4)	0.9 (0.8, 1.0)	0.9 (0.8, 1.0)	1.3 (1.2, 1.4)	0.4 (0.3, 0.4)	0.4 (0.4, 0.5)	0.7 (0.6, 0.8)
	**Girls**								
	**10–12 years**			**13–14 years**			**15–16 years**		
	**% (95% CI) 2006**			**% (95% CI) 2010**			**% (95% CI) 2014**		
**Healthy lifestyle behavior**	**2006**	**2010**	**2014**	**2006**	**2010**	**2014**	**2006**	**2010**	**2014**
Physical activity every day	21.0 (20.5, 21.4)	18.1 (17.6, 18.5)	20.1 (19.6, 20.5)	14.2 (13.8, 14.6)	12.8 (12.4, 13.2)	14.9 (14.4, 15.3)	10.6 (10.3, 11.0)	9.5 (9.2, 9.9)	10.7 (10.3, 11.0)
Screen time <2 h/day	35.6 (35.1, 36.2)	32.5 (32.0, 33.1)	44.3 (43.7, 44.8)	21.5 (21.0, 21.9)	20.3 (19.9, 20.8)	25.0 (24.5, 25.4)	20.2 (19.8, 20.7)	18.6 (18.2, 19.1)	19.7 (19.2, 20.1)
Fruit/vegetable every day	25.7 (25.2, 26.2)	28.8 (28.2, 29.4)	29.2 (28.6, 29.7)	22.3 (21.8, 22.8)	23.7 (23.3, 24.2)	24.7 (24.2, 25.2)	20.9 (20.5, 21.4)	22.6 (22.1, 23.1)	24.4 (23.9, 24.9)
Not drink	74.8 (74.3, 75.3)	81.7 (81.3, 82.2)	84.8 (84.4, 85.2)	52.4 (51.8, 53.0)	61.1 (60.6, 61.6)	67.7 (67.2, 68.2)	27.6 (27.1, 28.1)	33.0 (32.5, 33.6)	41.6 (41.0, 42.1)
Not smoke	98.3 (98.1, 98.4)	98.6 (98.4, 98.7)	98.9 (98.7, 99.0)	90.1 (89.7, 90.4)	91.3 (91.0, 91.6)	94.0 (93.7, 94.2)	75.1 (74.6, 75.6)	77.4 (76.9, 77.8)	83.5 (83.0, 83.9)
**Number of behaviors**									
None	2.3 (2.1, 2.5)	0.5 (0.4, 0.6)	0.3 (0.2, 0.4)	8.0 (7.7, 8.3)	4.8 (4.6, 5.0)	3.0 (2.8, 3.2)	13.1 (12.7, 13.4)	14.0 (13.6, 14.4)	9.7 (9.4, 10.1)
1. Behavior	15.4 (15.0, 15.8)	9.1 (8.7, 9.4)	6.3 (6.0, 6.6)	29.4 (28.8, 29.8)	21.8 (21.4, 22.3)	17.6 (17.2, 18.0)	38.7 (38.1, 39.2)	34.2 (33.7, 34.7)	30.3 (29.8, 30.9)
2. Behavior	35.0 (34.4, 35.5)	39.8 (39.3, 40.4)	34.3 (33.7, 34.8)	35.7 (35.2, 36.3)	42.0 (41.5, 42.6)	41.9 (41.3, 42.4)	31.5 (31.0, 32.1)	33.2 (32.6, 33.7)	36.7 (36.1, 37.2)
3. Behavior	31.5 (30.9, 32.0)	34.1 (33.6, 34.6)	38.0 (37.4, 38.5)	19.7 (19.3, 20.2)	23.0 (22.5, 23.4)	26.6 (26.2, 27.1)	13.2 (12.9, 13.6)	14.3 (14.0, 14.7)	17.7 (17.3, 18.1)
4. Behavior	13.0 (12.6, 13.4)	13.7 (13.3, 14.1)	17.3 (16.9, 17.8)	6.3 (6.1, 6.6)	7.3 (7.0, 7.5)	9.3 (8.9, 9.6)	3.2 (3.0, 3.4)	3.8 (3.6, 4.0)	4.9 (4.6, 5.1)
5. Behavior (healthy lifestyle)	2.8 (2.6, 3.0)	2.8 (2.6, 3.0)	3.8 (3.6, 4.0)	0.9 (0.8, 1.0)	1.1 (1.0, 1.2)	1.6 (1.4, 1.7)	0.3 (0.3, 0.4)	0.5 (0.4, 0.5)	0.7 (0.6, 0.8)

Table 3 presents the prevalence of adolescents with a healthy lifestyle (reporting all five health behaviors) in 2006, 2010, and 2014 by sex and for each country of HBSC data. Overall, for all countries, the healthy lifestyle increases between the years, except for boys from Finland and girls from Luxemburg. For boys, the highest values were observed in adolescents from Ireland (5.2%, 95% CI: 3.9, 6.4) and Norway (4.5%, 95% CI 2.2, 6.8). For girls, the highest values were observed in adolescents from Iceland (4.2%, 95% CI: 3.6, 4.7) and Ireland (4.2%, 95% CI: 3.3, 5.0). The highest values for boys and girls were from 2014. For boys, the worst values were from Romania (0.3%, 95% CI: 0.1, 0.6) in 2006 and Latvia (0.3%, 95% CI: 0.0, 0.5) and Estonia (0.3%, 95% CI: 0.0, 0.5)—both in 2010. For girls, the worst values were from Romania (0.2%, 95% CI: 0.0, 0.4) and Russia (0.2%, 95% CI: 0.1, 0.3)—both in 2006.

**Table 3:** Prevalence of adolescents with a healthy lifestyle (reporting all five health behaviors) in 2006, 2010, and 2014 by sex and for each country of HBSC data.

	**Boys**			**Girls**		
	**2006**	**2010**	**2014**	**2006**	**2010**	**2014**
**Country**	**% (95% CI)**	**% (95% CI)**	**% (95% CI)**	**% (95% CI)**	**% (95% CI)**	**% (95% CI)**
Austria	0.7 (0.4, 1.1)	2.2 (1.6, 2.9)	2.8 (1.9, 3.7)	1.7 (1.2, 2.3)	2.7 (2.1, 3.4)	3.4 (2.4, 4.3)
Belgium	1.4 (0.9, 1.9)	1.5 (0.9, 2.0)	2.1 (1.7, 2.5)	2.6 (1.9, 3.3)	1.6 (1.1, 2.2)	1.9 (1.5, 2.3)
Canada	2.1 (1.6, 2.7)	1.8 (1.5, 2.1)	3.4 (2.9, 3.9)	2.2 (1.6, 2.7)	2.2 (1.9, 2.6)	3.8 (3.3, 4.3)
Croatia	0.9 (0.5, 1.3)	0.5 (0.3, 0.8)	2.0 (1.4, 2.6)	1.3 (0.8, 1.7)	0.8 (0.5, 1.1)	2.1 (1.5, 2.7)
Czech Republic	0.7 (0.3, 1.0)	0.8 (0.4, 1.1)	0.9 (0.5, 1.3)	1.4 (0.9, 1.8)	1.0 (0.6, 1.5)	1.9 (1.3, 2.5)
Denmark	1.7 (1.2, 2.2)	0.9 (0.4, 1.3)	1.2 (0.6, 1.8)	3.0 (2.4, 3.7)	1.0 (0.6, 1.4)	1.4 (0.9, 2.0)
England	0.9 (0.5, 1.3)	1.4 (0.7, 2.1)	1.9 (1.3, 2.5)	1.2 (0.7, 1.7)	0.9 (0.5, 1.4)	2.3 (1.7, 3.0)
Estonia	0.4 (0.1, 0.7)	0.3 (0.0, 0.5)	1.1 (0.6, 1.5)	0.5 (0.2, 0.7)	0.8 (0.4, 1.2)	1.0 (0.5, 1.4)
Finland	1.5 (1.0, 2.0)	0.8 (0.4, 1.3)	0.7 (0.3, 1.1)	2.2 (1.6, 2.8)	1.4 (0.9, 1.9)	2.6 (1.8, 3.3)
France	0.9 (0.6, 1.2)	1.1 (0.7, 1.5)	1.3 (0.8, 1.8)	0.7 (0.4, 0.9)	0.8 (0.5, 1.2)	1.2 (0.7, 1.7)
Germany	1.0 (0.6, 1.3)	1.1 (0.7, 1.5)	1.4 (0.9, 1.9)	1.2 (0.8, 1.6)	1.4 (0.9, 1.9)	2.0 (1.4, 2.5)
Greece	0.4 (0.1, 0.7)	0.4 (0.1, 0.7)	0.5 (0.2, 0.8)	0.5 (0.2, 0.8)	0.6 (0.3, 0.9)	1.0 (0.6, 1.5)
Hungary	0.8 (0.4, 1.3)	1.5 (1.0, 2.0)	2.0 (1.3, 2.7)	0.8 (0.3, 1.2)	1.6 (1.1, 2.1)	2.6 (1.8, 3.3)
Iceland	2.2 (1.7, 2.6)	1.5 (1.2, 1.9)	3.8 (3.3, 4.4)	3.2 (2.7, 3.8)	2.8 (2.3, 3.2)	4.2 (3.6, 4.7)
Ireland	3.8 (2.9, 4.6)	2.3 (1.7, 3.0)	5.2 (3.9, 6.4)	3.7 (2.9, 4.6)	3.1 (2.3, 2.9)	4.2 (3.3, 5.0)
Israel	1.0 (0.5, 1.4)	1.0 (0.6, 1.5)	1.2 (0.5, 1.9)	0.8 (0.4, 1.1)	0.5 (0.2, 0.8)	0.7 (0.2, 1.2)
Italy	0.6 (0.2, 0.9)	0.5 (0.2, 0.8)	0.7 (0.3, 1.1)	0.7 (0.3, 1.1)	0.7 (0.3, 1.0)	0.7 (0.3, 1.1)
Latvia	0.5 (0.2, 0.9)	0.3 (0.0, 0.5)	1.0 (0.6, 1.5)	0.4 (0.1, 0.7)	0.6 (0.3, 1.0)	1.5 (1.0, 2.0)
Luxembourg	0.7 (0.3, 1.1)	2.6 (1.8, 3.3)	4.1 (2.9, 5.3)	1.6 (1.0, 2.1)	2.8 (2.2, 3.6)	2.3 (1.5, 3.1)
Macedonia	0.8 (0.4, 1.1)	1.1 (0.6, 1.5)	1.8 (1.2, 2.5)	1.2 (0.8, 1.6)	1.8 (1.2, 2.5)	3.3 (2.5, 4.1)
Netherlands	0.6 (0.3, 1.0)	0.7 (0.4, 1.1)	0.9 (0.5, 1.3)	0.9 (0.5, 1.3)	1.4 (0.9, 1.9)	1.2 (0.7, 1.7)
Norway	2.1 (1.5, 2.8)	0.4 (0.0, 0.8)	4.5 (2.2, 6.8)	2.2 (1.5, 2.9)	0.8 (0.2, 1.3)	2.5 (0.9, 4.1)
Poland	0.4 (0.1, 0.6)	0.5 (0.2, 0.9)	2.3 (1.6, 2.9)	0.8 (0.5, 1.2)	1.0 (0.5, 1.4)	2.5 (1.8, 3.2)
Portugal	0.5 (0.2, 0.9)	0.6 (0.2, 1.0)	1.9 (1.3, 2.5)	0.5 (0.2, 0.8)	0.5 (0.2, 0.8)	1.6 (1.0, 2.1)
Romania	0.3 (0.1, 0.6)	0.4 (0.1, 0.6)	1.0 (0.5, 1.5)	0.2 (0.0, 0.4)	0.8 (0.5, 1.2)	1.5 (0.9, 2.1)
Russia	0.4 (0.2, 0.6)	0.7 (0.4, 1.1)	1.6 (0.9, 2.2)	0.2 (0.1, 0.3)	0.7 (0.4, 1.0)	1.8 (1.2, 2.3)
Scotland	1.1 (0.7, 1.4)	0.9 (0.6, 1.2)	1.8 (1.2, 2.3)	1.1 (0.7, 1.5)	1.0 (0.6, 1.3)	2.0 (1.4, 2.5)
Slovakia	1.0 (0.5, 1.5)	0.7 (0.4, 1.1)	1.3 (0.8, 1.7)	0.7 (0.3, 1.1)	0.7 (0.3, 1.0)	2.1 (1.5, 2.7)
Slovenia	0.8 (0.5, 1.2)	0.8 (0.5, 1.1)	2.0 (1.4, 2.7)	1.8 (1.3, 2.3)	1.7 (1.2, 2.2)	2.7 (2.0, 3.3)
Sweden	2.2 (1.5, 2.9)	0.8 (0.4, 1.1)	1.2 (0.8, 1.5)	1.8 (1.2, 2.4)	1.2 (0.8, 1.6)	1.4 (1.0, 1.9)
Switzerland	2.2 (1.6, 2.9)	1.8 (1.3, 2.3)	3.6 (2.9, 4.4)	2.0 (1.4, 2.6)	1.8 (1.3, 2.2)	3.0 (2.4, 3.7)
Wales	0.5 (0.2, 0.9)	0.5 (0.2, 0.8)	1.0 (0.6, 1.4)	1.0 (0.6, 1.4)	0.9 (0.5, 1.3)	1.5 (1.0, 2.0)

*HBSC, Health Behavior in School-aged Children*.

## Discussion

This study aimed to provide data regarding the prevalence and trends of adolescents' healthy lifestyles (age 10–16 years) from 2006 to 2014 from 32 countries. Based on the results of this study, between 2006 and 2014, the prevalence of healthy lifestyles increased significantly for boys and girls. When age increased, the prevalence of adolescents with a healthy lifestyle decreased significantly for all years analyzed. The results show a difference between boys and girls regarding the behaviors that were more difficult to reach the guidelines for a healthy lifestyle. For boys, it was more difficult to eat fruit and vegetables every day and spend <2 h on screen, while for girls, being physically active 60 min daily was the main obstacle. On the other hand, not smoking followed by not drinking were the behaviors with the highest values for both boys and girls. Regarding adolescents' healthy lifestyle prevalence by country, the lowest values were observed in countries from eastern European countries (e.g., Romania, Latvia, Estonia, and Russia), and the highest values were from Nordic European countries (e.g., Norway and Iceland) and Ireland. Inequalities in health are well-known among European countries (20). Such analyses proved to be fundamental to identify trends concerning adolescents' healthy behaviors, as well as to designing programs that aim to improve adolescents' healthy behaviors. Although a slight improvement has been observed across time, yet the values are still low, which denote a concern, since these behaviors are related to the increase of vulnerability, such as in terms of chronic diseases in adulthood (21) and to the greater subjective health complaints during adolescence (6).

Regarding physical activity, our study showed a significant decreasing trend from 10- to 16-year-old individuals and it is always significantly less practiced by girls than boys across all ages. Physical activity was a behavior that showed no significant difference across time, without a tendency for improvement, in both boys and girls. Data from 146 countries including 1.6 million students aged 11–17 years, from 2001 to 2016, showed a sex difference regarding being physically active (22). Notably, this study shows that the percentage of insufficiently active boys, 77.6% (95% CI: 76.1, 80.4), is lower than that of girls, 84.7% (95% CI: 83.0, 88.2). Furthermore, a significant temporal decrease between 2001 and 2016 was observed for boys but not for girls. Overall, 81% (95% CI: 77.8, 87.7) of adolescents are insufficiently physically active (22). Those findings reinforce the need to implement effective strategies to advance with the World Health Organization recommendation to reduce physical inactivity by 10% by 2025 and 15% by 2030 (22). The benefits of being physically active during adolescence are well-documented, including improvements in cardiorespiratory and muscular fitness, bone and cardiometabolic health, and positive effects on weight status (23). Importantly, those benefits carry on from adolescence to adulthood.

Our study found that boys and girls aged 10–14 years increase their healthy behavior regarding spending <2 h with a screen. Nonetheless, among boys and girls aged 15 and 16 years, no significant difference was observed from 2006 to 2014. We analyzed screen time as a period including television watching, playing videogames, and computer time. Other studies that analyzed this behavior in more detail found a decrease in time spent watching TV in recent years and an increase in time spent on computer (24–26). With increasing age, there is a greater need to use the computer as a pedagogical resource, which may, in part, justify the increase in screen time found in our study. Besides that, for all years and all ages, boys spent significantly more hours in front of a screen than girls. Another study also found sex differences and argued that boys are more interested in computer games than girls (24).

Concerning fruit and vegetable consumption, our analyses show a positive and significant trend to increase the consumption from 2006 to 2014. A significant sex difference was observed, with girls healthier than boys regarding fruit and vegetable consumption in all years and ages. Other study found that only 8% of European adolescents met the international recommendations of fruit and vegetable consumption (27). Our analyses did not consider how many portions they ate, just whether adolescents eat fruit and vegetables daily or not. International recommendations establish five servings per day (28). In future research, it is thus important to look for the determinants of fruit and vegetable intake, such as sex, age, socioeconomic position, preferences, parental intake, and home availability and accessibility (29).

Early-onset alcohol consumption exposure is associated with various cognitive and other functional deficits (30). Our results showed a positive and significant trend to decrease the consumption (any use, independently of frequency, or quantity), among boys and girls, across time. Our results corroborate a sex difference (boys consume more than girls) and an increase with age also observed in other studies (31, 32). Among adolescents, the social environment influences health behaviors mainly related to alcohol and smoking. Networks of peers were found to have similar risky health behaviors when it comes to smoking, drinking, and cannabis use (33). Regarding smoking behavior, our data showed a significant and positive trend to improvement among boys and girls with 13–14 years and 15–16 years. For 10–12 years, no changes were observed. Similar to alcohol consumption, boys smoke significantly more than girls. This result reinforces the necessity of socio tobacco policy, since it is associated with a lower likelihood of smoking among adolescents (34).

This study has some limitations. The study included information from school adolescents. School adolescents may vary from others of the same age, as adolescents in school may be more likely to come from advantaged backgrounds that have access to more healthy behaviors. Data were self-reported, which can lead to some bias. Also, the recruitment process was the responsibility of each country enrolled in the study, so possible sampling bias should be considered. Fruit and vegetable consumption did not consider how many portions they ate, which would be important according to prescribed guidelines (28). The alcohol and tobacco use was dichotomized and did not assess quantity consumed per occasion and did not assess other substance use (e.g., cannabis). The HBSC survey did not assess sleep information, and it would be an important measure for adolescents' healthy lifestyles (35). Also, the comparisons are descriptive; no formal statistical test of differences between groups was conducted (i.e., no formal control for multiple testing). These shortcomings might be addressed in future research.

## Conclusion

Overall, our study showed a slight trend to an improved healthy lifestyle among adolescents, although much more has to be done. The concern should be mainly about physical activity and screen time—both behaviors that did not present a significant trend of improvement. A joint effort from multiple areas of knowledge must be made to improve adolescent health policies, since lifestyles in adolescence play an important role for the development of vulnerability and health in later life.

## Data Availability Statement

Publicly available datasets were analyzed in this study. This data can be found here: https://www.uib.no/en/hbscdata.

## Ethics Statement

The studies involving human participants were reviewed and approved by the data used in the present study were derived from the HBSC international database. Each participant country is responsible for researching under their ethical guidelines, consequently, consent to carry out the research was given by school administrators in each country. Written informed consent to participate in this study was provided by the participants' legal guardian/next of kin.

## Author Contributions

PM and AM conceived and designed the analysis, performed the analysis, and drafted the article. MM, AI, GF, ÉG, ML-F, and MP revised it critically for important intellectual content. AM conceived and designed the analysis, performed the analysis, and revised it critically for important intellectual content. All authors contributed to the article and approved the submitted version.

## Conflict of Interest

The authors declare that the research was conducted in the absence of any commercial or financial relationships that could be construed as a potential conflict of interest.
